# Development of the High Sensitivity and Selectivity Method for the Determination of Histamine in Fish and Fish Sauce from Vietnam by UPLC-MS/MS

**DOI:** 10.1155/2020/2187646

**Published:** 2020-06-17

**Authors:** Quang Hieu Tran, Thanh Tan Nguyen, Kim Phuong Pham

**Affiliations:** ^1^Chemistry Division, Basic Sciences Department, Saigon Technology University, 180 Cao Lo, Ward 4, District 8, Ho Chi Minh City 700000, Vietnam; ^2^Saigon STC Hi-Tech Analytical Center, Ho Chi Minh City 700000, Vietnam

## Abstract

A selective, sensitive, and rapid method by using ultraperformance liquid chromatography-tandem mass spectrometry (UPLC-MS/MS) for the determination of histamine in fish and fish sauce was developed. The optimal conditions of liquid chromatographic separation and mass spectroscopy of histamine have also been investigated. The linear ranges of the method were 20.0 ÷ 1000 ng/mL, and the corresponding correlation coefficient was 0.9993. Mean recoveries of the analyte at three spike levels (low, medium, and high) were within the range of 98.5% ÷ 102.5% (*n* = 7). The limit of detection (LOD) and limit of quantification (LOQ) values were 3.83 and 11.50 ng/mL for the fish sauce sample and 4.71 and 14.12 ng/mL for the fish sample, respectively. The influence of the matrix effect on the accuracy, repeatability, and recovery of the method was negligible. The recommended method was applied to determine the content of this substance in 21 fish sauce samples and 4 kinds of fish samples, which were collected from Ho Chi Minh City, Vietnam, in 2019.

## 1. Introduction

For a long time, the content of histamine in fish and fishery products as well as its poisoning has attracted a lot of attention from scientists around the world. This compound is formed during the breakdown of the amino acid histidine which follows two ways. The major route of catabolism of histidine passes through its conversion to glutamic acid, which begins with the degradation of histidine to urocanic acid by the action of the enzyme histidase. The glutamate product is converted to alpha-ketoglutarate, which is an intermediate in the citric acid cycle (Krebs cycle). The second way is decarboxylation (loss of COO-) for the action of the enzyme histidine decarboxylase to form histamine [1, 2]. Scombroid syndrome/histamine poisoning occurs worldwide, and the number of cases is increasing, in spite of the improved knowledge on seafood safety [3]. The symptoms of histamine poisoning generally resemble the symptoms encountered with IgE-mediated food allergies. The symptoms include nausea, vomiting, diarrhea, an oral burning sensation or peppery taste, hives, itching, red rash, and hypotension [4]. The onset of the symptoms usually occurs within a few minutes after the ingestion of the implicated food, and the duration of symptoms ranges from a few hours to 24 h [1]. Due to the toxicity of histamine, the content of this substance has been strictly controlled. According to Codex 302-2011, histamine is considered to be a hazard in fish sauce, and the contents of this compound must not exceed 400 mg/kg [5, 6]. The Commission Regulation (EC), no. 1019/2013, recommended that the level of histamine must not be exceeded than 400 mg/kg in fish sauce and 200–400 mg/kg in the fishery products [7]. The European Union and the United States Food and Drug Administration (FDA) have fixed 100 and 50 mg/kg for the maximum level of histamine in fish and fishery products, respectively [8, 9].

Fish sauce, a fundamental ingredient used in many Southeast Asian dishes, a dipping condiment, is gradually gaining popularity worldwide [10]. Fish sauce is a clear brown liquid produced by spontaneous fermentation of diverse fish such as anchovies, sardines, and menhaden [11]. These fishes typically possess high levels of free histidine and other amino acids. The quantity of histamine produced in traditional fish sauce is related to the free histidine content in the raw materials. During fermentation, protein hydrolysis is caused by endogenous proteinases in the fish muscle and digestive tract as well as proteinases produced by halophilic bacteria [2, 12]. The species most commonly used for fish sauce production is Indian anchovy (*Stolephorus* spp.). Anchovies are normally caught and kept on board. Producers with good manufacturing practices would mix fish with salt after the catch, which retards histamine formation. However, some keep fish without salt for up to 8 hours before landing and transport to a factory in an open container without a proper cooling system [13]. Due to the nature of raw materials and the production methods for traditional fish sauce, high levels of histamine are found in many samples [14, 15]. There are more than 2,800 fish sauce production facilities in Vietnam, which has been producing more than 200 million liters per year, worth over VND 4,800 billion. However, Vietnam's fish sauce exports account for only about 3–5% of the production. The leading cause of the current low export volume is that there are no well-established brands of Vietnamese fish sauce overseas. Besides, another important issue is that traditional Vietnamese fish sauce often encounters technical barriers with some international quality standards, such as histamine level [16]. Currently, histamine has been detected by many methods, such as electrophoretic [17], thin layer chromatography [18], nuclear magnetic resonance (NMR) [19], photoluminescence CdTe quantum dots [20], colorimetry [21, 22], enhanced Raman spectroscopy (SERS) [23], electrochemical [24], nanogold particles (AuNPs) [25], HLPC-UV [14, 25, 26], LCMS/MS [26], UHPLC-Q/TOF-MS [11], or UHPLC-MS/MS [27]. Presently, the standard procedure for histamine analysis by UPLC-MS/MS has not been officially announced in Vietnam. Therefore, in this work, we have developed the analysis method to shorten the analysis time and increase the sensitivity and selectivity based on UPLC-MS/MS. Thereby, we hope to provide a novel analytical method for testing the presence of histamine in fish sauce and fishery products in Vietnam.

## 2. Materials and Methods

### 2.1. Chemicals and Reagents

All reagents were of analytical grade. The histamine reference standard was purchased from Merck (Darmstadt, Germany); acetonitrile (ACN) and formic acid (FA) were supplied from Sigma-Aldrich (Germany). Methanol was of HPLC grade and acquired from J. T. Baker (Phillipsburg, USA).

### 2.2. Instrumentation

The method development and validation was performed on an ultrahigh performance liquid chromatography (UPLC) system including the column oven and thermostat autosampler (Ultimate 3000, Thermo Fisher Scientific, Bremen, Germany) in combination with the Waters.

TQD tandem quadrupole mass spectrometer, operated in the electrospray positive ion mode with selected reaction monitoring (SRM), was used for data acquisition.

### 2.3. Standard Solutions

The stock standard solution of 1.0 mg/mL of histamine was prepared in ACN at a concentration of 1.0 mg/mL each. A working solution was prepared immediately before use by diluting 0.1 mL of the stock standard solution to 10 mL ACN to obtain a solution having a known concentration of 0.01 mg/mL. All stock solutions were kept refrigerated (2–8°C) when not in use.

### 2.4. Sample Preparation

#### 2.4.1. Fish Samples

Fish samples were collected from various commercial fish markets in Ho Chi Minh City. Each sample was placed individually in a plastic bag, kept in an icebox, and carried to the laboratory of Saigon STC Hi-Tech Analytical center, Ho Chi Minh City. For the examination, a representative fish sample (50 g) was chopped into small slices and finely ground with a blender to homogenize it before extraction. Then, 5.0 ± 0.1 g of the fish sample was transferred into a 50 mL centrifuge tube, added precisely 25 mL of MeOH, vortexed for 2.0 mins, and sonicated in an ultrasonic bath for 20 mins. The solution was transferred into a 50 mL volumetric flask, and volume was makeup with MeOH. Then, this solution was filtrated through a 0.45 *μ*m Whatman membrane. The solution of the sample has been diluted in DI water (the dilution factor depends on the amount of histamine in the sample) and was filtered through a 0.22 *μ*m filter into a 1.5 mL amber LC vial. The volume of 5.0 *μ*L of the sample solution was injected into the UPLC-MS/MS system via the autosampler at the optimum experimental conditions.

#### 2.4.2. Fish Sauce Samples

Fish sauce samples, including 10 industrial fish sauce (IFS) and 10 traditional fish sauce (TFS), were collected from the local markets and supermarkets in Ho Chi Minh City, Vietnam. The procedure for sample preparation is as follows: 1.0 mL of fish sauce was drawn accurately into the 50 mL volumetric flask, dissolved, and made up to the mark with MeOH. Then, 0.5 mL of this solution was transferred to a 10 mL volumetric flask, dissolved and made up to volume with DI water, and filtered through a 0.45 *µ*m filter. The solution of the sample was diluted in DI water (the dilution factor depends on the amount of histamine in the sample) and was filtered through a 0.22 *µ*m filter into a 1.5 mL amber LC vial. The analysis was performed on UPLC-MS/MS, as described in Section 2.4.1.

### 2.5. For Liquid Chromatographic Conditions

For liquidchromatographic separation, the HILIC Silica 3 *µ*m, 2.1 × 50 mm, Atlantis column was applied for the separation of target analytes at the temperature of 40°C. The binary mobile phases were ACN 100 mM + HCOOH (*A*) and DI + 0.5% HCOOH (*B*). The mobile phase program for the loading pumps is shown in Table 1. The flow rate was constantly kept at 0.3 mL/min during the whole chromatographic analysis process. Both samples and standard solutions were kept at 10°C in the sample tray. A 5.0 *µ*L of the standard or samples was injected into the UPLC-MS/MS system via an autosampler. The needle and the sample loop in the autosampler were washed triplicate, using the mixture of ACN and deionized water (1 : 1, v : v).

### 2.6. MS Conditions

For finding the optimal conditions of MS, selected reaction monitoring (SRM) spectra obtained in the positive ion mode were applied to recognize the specified analyte. The ion source parameters were optimized as follows: ionizing source: H-ESI (+), spray voltage: 3500 V, vaporizer temperature: 300°C, ion transfer tube temperature: 300°C, sheath gas: 40 arb, aux gas: 5 arb, and CID gas: 2 mTorr. TraceFinder 3.3 software was used for calculating the peak areas and peak area ratios.

### 2.7. Method Validation

#### 2.7.1. Linearity

For evaluating the linearity, a series of reference standard solutions of histamine were prepared in the range of concentrations of 20.0, 50.0, 150.0, 200.0, 500.0, and 1000.0 (ng/mL). At each level, the standard solutions were measured with triplicate. Based on the plotting of the peak area ratio to the internal standard versus the spiked concentration, the calibration curve equation was calculated.

#### 2.7.2. Determination of LOD and LOQ

Limit of detection (LOD) and limit of quantification (LOQ) are two important performance features in method validation. LOD and LOQ, strictly related to the magnitude of noises in the measurement system, could be determined in different ways. In this work, these values were calculated by the formula LOD = 3SD and LOQ = 3LOD according to [28, 29].

#### 2.7.3. Matrix Effect (ME)

The evaluation of the matrix effect was conducted by using an experimental model of Matuszewski et al. [30]. The matrix effect during validation of the developed method was examined by measurement of the analytical signal of histamine in the postextraction spiked solution and that of the histamine standard in a neat solution. The experiment was carried out with two groups of pooled samples. The first group was prepared by mixing five fish sauce samples, and the second group was formed by mixing four fish samples (sardines, anchovy, menhaden, and mackerel). The sample preparation was performed in Section 2.4.1. The final solution (after sample extraction) was spiked in three levels of 50, 200, and 1000 ppb of standard histamine. The effect of the sample matrix was determined by the following equation:

(1) $$\begin{array}{c} \text{ME}\left(\%\right)=\frac{B}{A}\times 100, \end{array}$$

where *A* is the chromatographic peak area of the standard in neat solution, *B* is the peak area of the standard spiked into sample solution after extraction by methanol.

#### 2.7.4. Recovery and Precision

For examining recovery and precision, the experiment was carried out with two kinds of samples (fish and fish sauce). A series of the example was spiked histamine samples at 214, 428, and 856 mg/L. The solution was diluted 20 times in DI water. The analysis was performed on UPLC-MS/MS, as described in Section 2.4.1. At each level, the test was measured seven times for calculating SD, *R*%, and RSD%.

## 3. Results and Discussion

### 3.1. Chromatographic and MS Conditions

#### 3.1.1. Chromatographic Conditions

**Figure 1:**
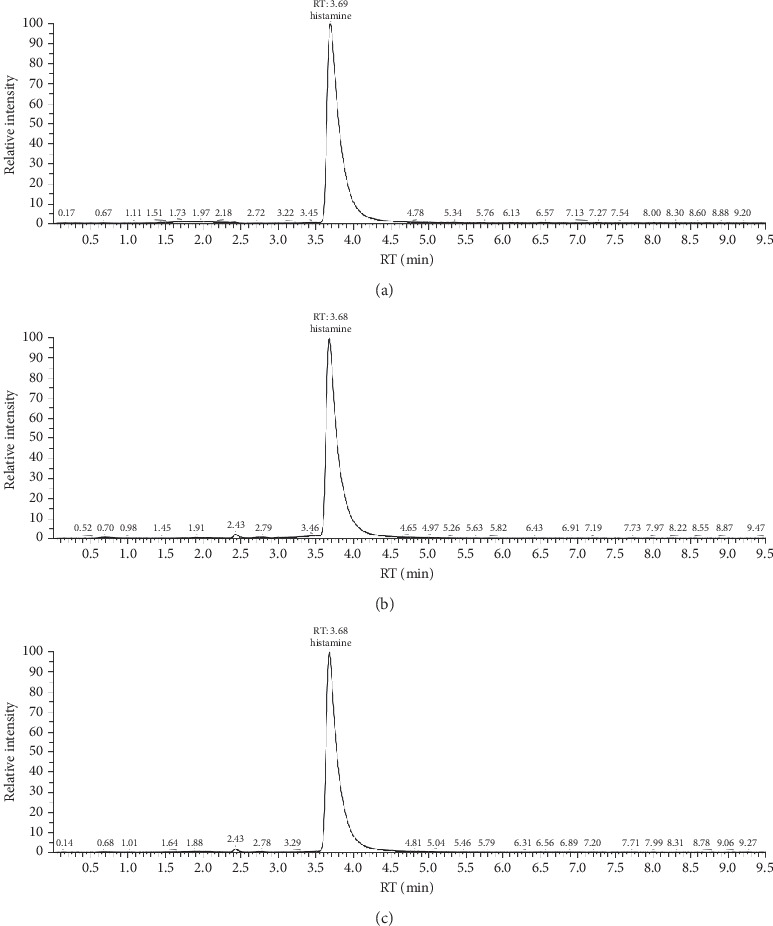
Representative UPLC-MS/MS chromatograms of histamine at 50.0 ng/mL of concentration: (a) in ACN blank, (b) in fish sauce sample solution, and (c) in fish sample solution.

The conditions for UPLC chromatography have been established with the HILIC Silica 3 *µ*m, 2.1 × 50 mm, Atlantis column, mobile phases A (MeOH 5 mM HCOONH_4_ + 0.1% FA) and B (H2O 5 mM HCOONH_4_ + 0.1% FA), and 5.0 *µ*L injection volume. The chromatogram of the standard solutions under selected conditions indicates the retention time of 3.69 mins for the neat solvent and 3.68 for both fish and fish sauce sample solutions.  Figure 1 indicates that there is no significant difference in the chromatograph of the standard histamine neat solvent and in the fish sauce sample solution at the same concentration. The retention time and the intensity of the analyte in both samples are related. This phenomenon implies that the effect of the sample background on the retention time is not notable.

#### 3.1.2. MS Parameters

**Figure 2:**
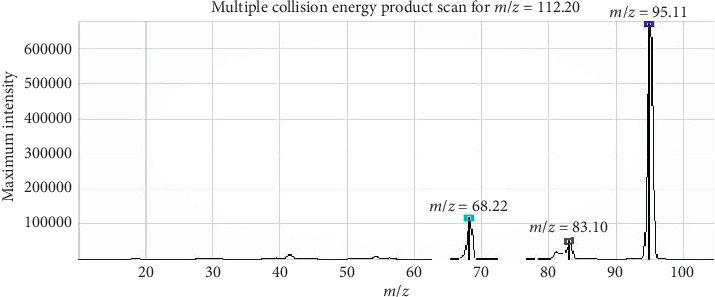
The maximum intensity of product scan for histamine in the SRM mode.

Table 2 and Figure 2 describe the mass spectrometry parameters of histamine and its fragmented ions. The precursor ion shows 112.2 of *m*/*z*; the product ions are 68 of *m*/*z*, 83 of *m*/*z*, and 95 of *m*/*z*, respectively. The collision energy for the ions is 23.0 V (*m*/*z* 68), 16.0 V (*m*/*z* 83), and 15.0 V (*m*/*z* 95), respectively. The *m*/*z* 95 ion is selected for the quantitation analysis. This result is quite similar to previous research studies [14, 24].

### 3.2. Method Validation

#### 3.2.1. Linearity

**Figure 3:**
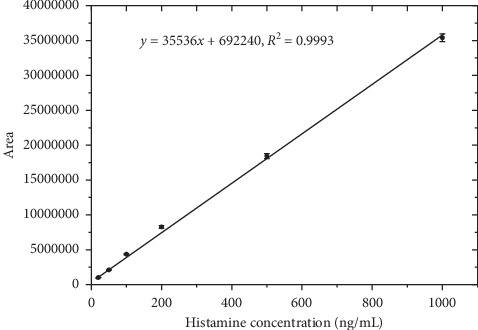
The graph of peak area vs. histamine concentration.

As present in Section 3.1, the matrix effects on two samples (fish and fish sauce) are not significant on the retention time and the intensity of the method. For simplifying the process, we have built the calibration curve in the pure solvent. The calibration curve in Figure 3 is linear over the range of 20.0 to 1000.0 ng/mL. The calibration equation obtained from the proposed method was *y* = 35536*x* + 692240. The least-squares regression exhibited an excellent correlation coefficient of 0.9993. The relative standard deviation of each point (*n* = 3) was less than 3.0%.

#### 3.2.2. LOD and LOQ

As can be seen in Table 3, LOD and LOQ of the validated method for fish sauce samples and fish samples are 3.83 and 11.52 and 4.71 and 14.12 ng/mL, respectively. Therefore, the developed method was suitable for direct analysis of this substance in fish and fish sauce samples according to the EC [7] and the USFDA [8].

#### 3.2.3. Selectivity

Endogenous sources of interference were not observed at the retention time of the analyte in Figure 1. This phenomenon indicates that the effect of the sample background on the retention time is not significant as compared with the sample blank. These values suggest that there is a minimum effect of the sample background on ion suppression, ion enhancement, and the retention time of analytes. These data also prove that the selectivity of the method for the determination of histamine in real samples is excellent.

#### 3.2.4. Matrix Effect

As can be seen, in Table 4, at three sample backgrounds with different fat contents, ME values in the range from 98.23% to 104.01% were in an acceptable range [30] (from 80 to 120%). The relative standard deviations at different concentrations are suitable values. These values show that the influence of the sample background on the selectivity and the recovery of measurement is negligible. This phenomenon could be explained by the excellent separation ability of the HILIC Silica column and the high sensitivity of the MS/MS probe. Compared with other methods for the determination of histamine with complex extraction, this method significantly shortens the analysis time, as well as reduces the analysis cost and the amount of solvent released after the sampling process.

#### 3.2.5. Recovery and Precision

In this test, the levels of 214, 428, and 856 mg/L, approximately to the level of 0.5, 1.0, and 2.0 times compared with the EC threshold prescribed for histamine standards in fish sauce, were chosen to spike. Similarly, the levels of 25, 50, and 100 mg/kg equivalent to 0.5, 1.0, and 2.0 times with the EC regulation threshold for histamine standards in fish samples and fish products were selected for the determination of recovery, accuracy, and repeatability. As presented in Table 5, the recovery of the histamine detection method at three concentrations lies from 100.86% to 116.43% (for the fish sauce sample) and from 95.14% to 107.21% (for the fish sample). These values are consistent with the requirements of the AOAC [31] and similar to previous research studies [26, 32, 33]. Thus, the method in this work has a high recovery efficiency and can be applied to real sample analysis. The intraday repeatability was within the acceptable range, ranging from 1.61 to 2.52% of RSD, whereas interday repeatability ranges from 2.34 to 10.21% of RSD. Similar RSD values are found in the fish sample background. The observed RSD values for the precision study indicate that this method is sufficiently precise for routine analysis.

For illustration, the sensitivity of the developed method by UPLC-MS/MS, a comparison with the previously published methods for histamine determination is given in Table 6. The developed method provides higher sensitivity in comparison with published methods. The sensitivity of this method is similar to the selectivity of the cITP-CZE-COND method.

### 3.3. Application to Real Samples

**Figure 4:**
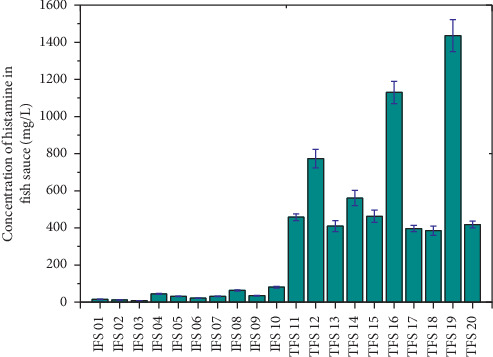
Histamine concentration of fish sauce; values are expressed as the mean ± SD (*n* = 3). Error bars represent the standard deviation of three replicates.

The proposed method has been applied to analyze 21 fish sauce samples. As shown in Table 7 and Figure 4, the group of industrial fish sauce (IFS 00 to IFS 10) contains histamine with the content from 8.0 to 80.9 mg/L. In general, the histamine content in 11 types of industrial fish sauce analyzed was the equally low allowable threshold of the FDA [8] and below of the EC [7]. The cause may be due to the closed industrial fish sauce production process and the raw fish that has been salted or frozen immediately after catching. Therefore, the conversion rate from histidine to histamine is less. Also, it is possible that, in industrial products, the content of pure fish sauce is low, and the manufacturer has added spices and flavorings to create industrial fish sauce. Therefore, the histamine content may be much lower than that of traditional fish sauce. Meanwhile, in the traditional fish sauce group (TFS 10 to TFS 20), the histamine content ranges from 385.6 to 1436.4 mg/L. These values are higher than the FDA's allowable threshold. The histamine content in traditional fish sauce in Vietnam is quite similar to other countries in the region, such as Thailand [12, 14], Malaysia [15], and China [11]. In fact, in the past, people in Asian countries have used traditional fish sauce with high levels of histamine content. However, histamine poisoning from fish sauce is very rare, because the amount of fish sauce in the daily meal is not much. However, for traditional fish sauce to penetrate the market of European countries or the US, manufacturers, scientists, and managers must find an explanation for the histamine content.

Four raw fish samples commonly used to produce fish sauce have been analyzed.  Table 8 indicates that the histamine content in these samples ranges from a fairly wide range, with the highest in mackerel, reaching 65.4 mg/kg. The histamine content depends on the type and freshness of fish. The more the fish becomes spoiled, the higher the content of histamine will be [37]. The histamine content of fish samples analyzed was quite similar to the results of the previous report in Vietnam [38] and other countries [20, 26]. We have also applied the AOAC standard procedure with HPLC-FDA equipment to determine the level of histamine in fish sauce and fish samples. The results illustrated in Tables 7 and 8 also showed that both methods perform harmonious results.

## 4. Conclusions

In this work, we have developed and evaluated a new analytical method that has excellent sensitivity, selectivity, and recovery. The method described here shows the promise of highly selective and sensitive quantification of histamine in fish sauce and fish samples. The HPLC-FDA method has also been used for comparative analysis, showing that the proposed method has similar results but with shorter time and more straightforward sample processing. The study of real samples has also shown that the histamine content in traditional fish sauce, produced by individual establishments, is much higher than that of the EC and FDA standards. Hopefully, we have contributed a new analytical technique to assess and control histamine content in fish, fishery products, food, and pharmaceuticals.

## Figures and Tables

**Table 1:** The mobile phase gradient program.

Time (min)	Flow rate (mL/min)	*A* (%)	*B* (%)
0	0.3	90	10
4.2	0.3	40	60
4.3	0.3	90	10
9.5	0.3	90	10

**Table 2:** Mass parameters of histamine and its products in the SRM mode.

Compound name	Polarity	Precursor (*m*/*z*)	Product (*m*/*z*)	Collision energy (V)
Histamine	Positive	112.2	68.222	23.0
Histamine	Positive	112.2	83.169	16.0
Histamine	Positive	112.2	95.111^*∗*^	15.0

^*∗*^Quantitative ion.

**Table 3:** The detection limit and quantitation limit of the UPLC-MS/MS method.

Replica (*n* = 7)	Fish sauce samples		Fish samples	
	Spiked (ng/mL)	Found (ng/mL)	Spiked (ng/mL)	Found (ng/mL)
1	20.00	19.21	20.00	18.12
2	20.00	21.32	20.00	22.13
3	20.00	21.20	20.00	21.40
4	20.00	18.70	20.00	18.36
5	20.00	20.15	20.00	21.14
6	20.00	21.10	20.00	21.20
7	20.00	22.31	20.00	20.90
Mean		20.57		20.46
SD		1.28		1.57
LOD = 3 SD (ng/mL)		3.83		4.71
LOQ = 3 LOD (ng/mL)		11.50		14.12

**Table 4:** ME (%) of the method at different matrix samples; the standard amount of histamine was spiked in three levels of 50, 200, and 1000 ppb after sample preparation.

Samples	Concentration of histamine in final sample solution (ng/mL)	Spiked (ng/mL) (*n* = 3)	ME (%)	RSD (%)
Pooled fish sauce	531.4	50	100.87	2.31
		200	104.01	1.67
		1000	101.34	2.24

Pooled fish	55.8	50	101.42	2.44
		200	102.07	0.74
		1000	98.23	1.21

**Table 5:** Recovery, intraday repeatability (day 1), and interday repeatability assay (day 3, day 5, and day 7) measured as %RSD of spiked fish sauce and fish samples (1.0 mL of fish sauce or 1.0 g of the fish sample, 50 mL of sample volume, and 20 times of dilution factor).

Samples	Concentration of histamine in the sample (ng/mL)	Spiked concentration (mg/L)	Recovery, *R* (%)	Repeatability (intraday), RSD (%)			
				Day 1	Day 3	Day 5	Day 7
Fish sauce, TFS 18	385.6	214	109.21	1.83	2.34	3.51	6.71
		428	100.86	2.55	3.22	8.34	4.29
		856	116.43	1.81	2.44	6.65	10.21

Mackerel fish	64.5	25	105.32	2.35	3.12	3.59	6.71
		50	95.14	3.49	3.67	6.15	6.91
		100	107.21	2.92	3.13	7.14	11.52

**Table 6:** The comparison of the developed UPLC-MS/MS method with previously published methods for the histamine analysis.

Methods	LOD (ng/mL)	LOQ (ng/mL)	Recovery (%)	References
UHPLC-HR-MS	100	300	—	[34]
cITP-CZE-COND	4.0	12.0	91 ± 9	[17]
IRP-HPLC	1000	3000	86.00	[35]
Colorimetric	5000		>91	[21]
UPLC-FLD	5.5	15.6	—	[34]
HPLC-UV	130	450	91 ÷ 115	[15]
UHPLC-MS/MS	3.38	11.5	100.1 ÷ 116	This work

**Table 7:** The concentration of histamine in fish sauce samples.

	Developed method (UPLC-MS/MS)	Compared method (HPLC-PDA)^1^
	Mean ± SD (mg/L)	Mean ± SD (mg/L)
IFS00	Not found	Not found
IFS 01	15.1 ± 1.1	16.2 ± 1.2
IFS 02	12.4 ± 0.8	13.5 ± 1.6
IFS 03	8.0 ± 0.5	7.2 ± 0.6
IFS 04	45.2 ± 2.2	48.3 ± 3.1
IFS 05	32.0 ± 1.3	35.2 ± 1.8
IFS 06	21.5 ± 1.2	24.3 ± 1.9
IFS 07	31.2 ± 1.4	28.1 ± 2.3
IFS 08	63.1 ± 3.2	66.3 ± 2.9
IFS 09	35.2 ± 2.4	38.6 ± 2.7
IFS 10	80.9 ± 5.0	87.4 ± 6.0
TFS 11	458.0 ± 18.3	432.0 ± 31.3
TFS 12	773.4 ± 50.5	761.4 ± 46.2
TFS 13	410.3 ± 30.5	391.6 ± 31.7
TFS 14	561.5 ± 41.6	589.3 ± 34.2
TFS 15	463.6 ± 33.2	432.4 ± 26.8
TFS 16	1130.1 ± 60.9	1093.4 ± 72.6
TFS 17	397.3 ± 17.6	410.4 ± 16.9
TFS 18	385.6 ± 25.4	374.3 ± 22.7
TFS 19	1436.4 ± 86.5	1501.3 ± 79.6
TFS 20	418.6 ± 18.3	424.8 ± 21.2

*Note.* ^1^Compared method was carried out by using the Waters 2996 HPLC-PDA instrument with column C18 (Zorbax Eclipse XDB-C18, 5 mm × 4.6 mm × 250 mm, Agilent) and followed the standard protocol of the Ministry of Science and Technology of Vietnam [22] and AOAC [36]. Each value is expressed as mean ± SD (*n* = 3).

**Table 8:** The concentration of histamine in fish samples.

	Developed method (UPLC-MS/MS) (mg/kg)	Compared method (HPLC-PDA) (mg/kg)
Frozen sardines *(Sardinella tawilis)*	16.4 ± 0.8	18.3 ± 1.2
Frozen anchovy *(Engraulidae)*	13.5 ± 0.9	12.1 ± 0.8
Frozen menhaden *(Brevoortia tyrannus)*	6.3 ± 0.7	7.5 ± 0.6
Frozen mackerel (*Rastrelliger kanagurta)*	64.5 ± 1.6	58.5 ± 3.2

*Note.* Each value is expressed as mean ± SD (*n* = 3).

## Data Availability

The data used to support the findings of this study are available from the corresponding author upon request. The MS optimization, some sample chromatographs, recovery performance calculations, and calibration curves are presented in Supplementary Information (available here).

## Supplementary Materials

Supplementary Materials S1: compound optimization report. S2: UPLC-MS/MS chromatograms of histamine in some of fish sauce and fish samples. S3: data for determination of *R*% and repeatability (fish sauce). S4: compound calibration report.
