# Supplementary material for: Development of the High Sensitivity and Selectivity Method for the Determination of Histamine in Fish and Fish Sauce from Vietnam by UPLC-MS/MS
Source: Int J Anal Chem. 2020 Jun 17;2020:2187646. doi: 10.1155/2020/2187646 (PMC7317331; doi:10.1155/2020/2187646)
Supplement: Supplementary Materials — S1: compound optimization report. S2: UPLC-MS/MS chromatograms of histamine in some of fish sauce and fish samples. S3: data for determination of R% and repeatability (fish sauce). S4: compound calibration report. [file 2187646.f1.docx]

**S1.Compound Optimization Report**

**S2. UPLC-MS/MS chromatograms of the Histamine in some of Fish sauce and fish samples.**

**IFS 00**


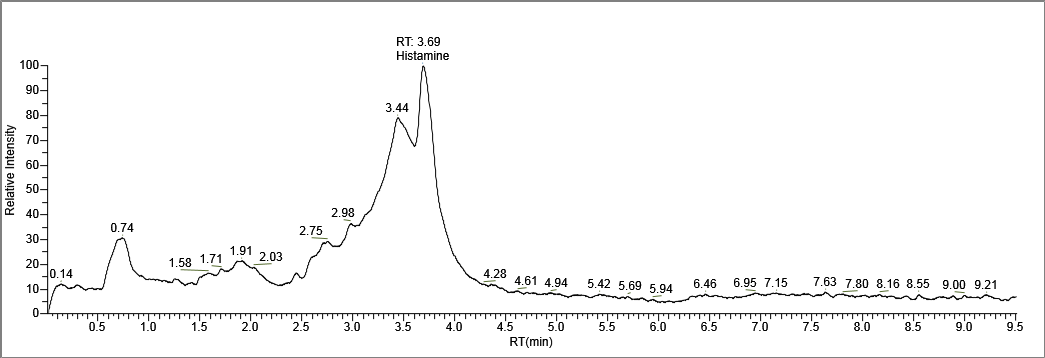


**IFS 01**


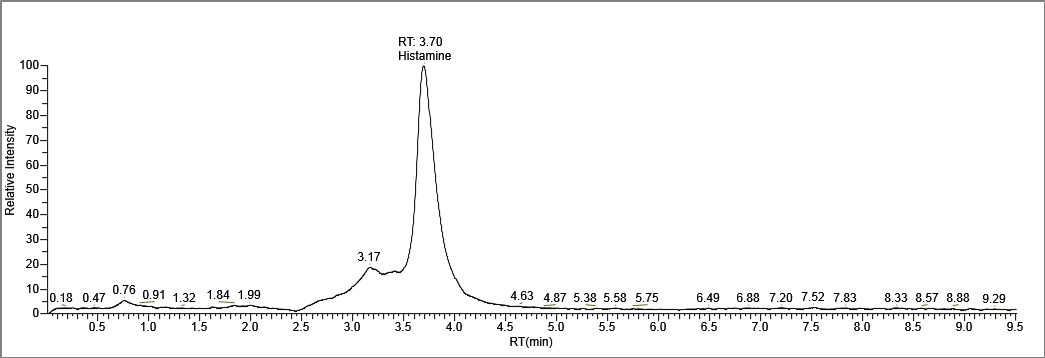


**IFS 02**


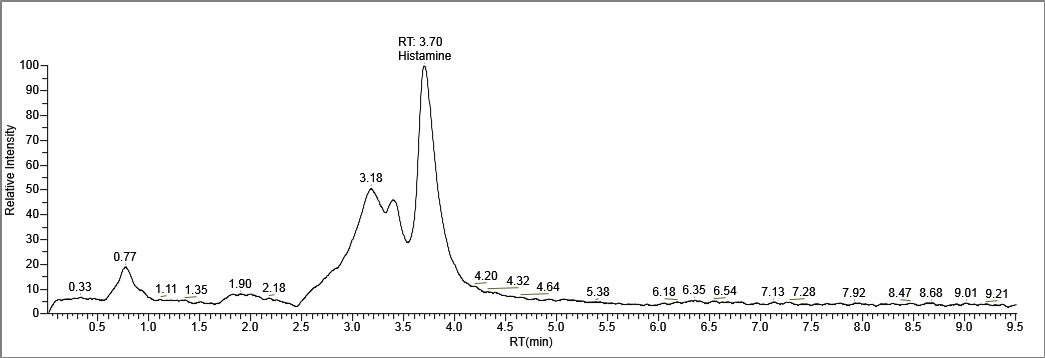


**TFS12**


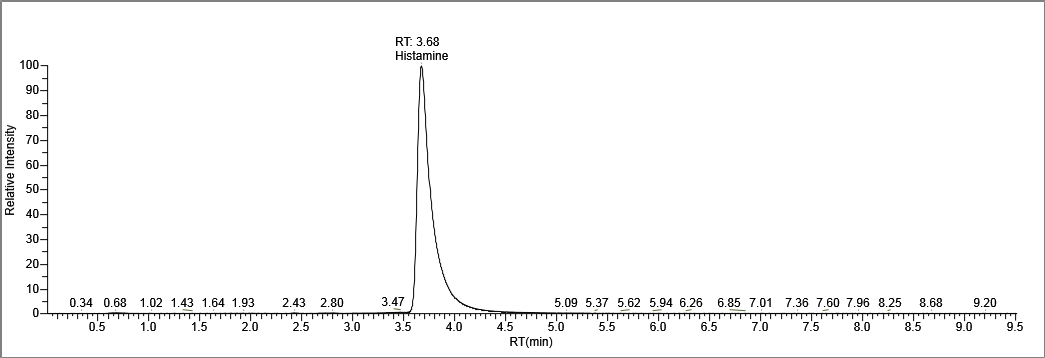


**TFS 18**


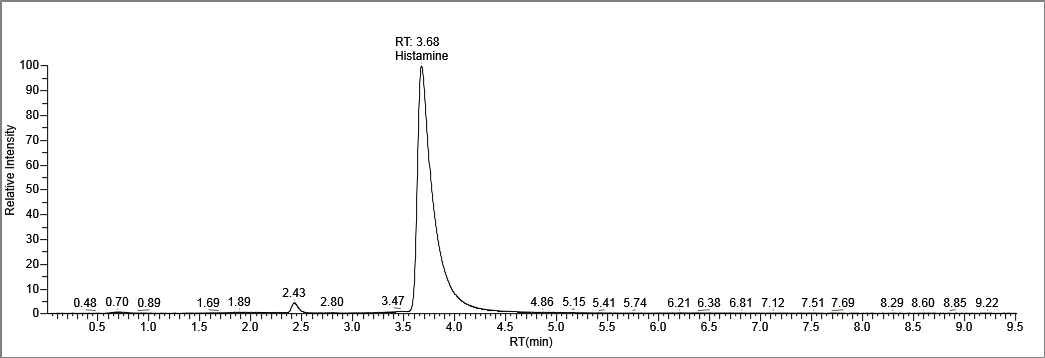


**TFS 19**


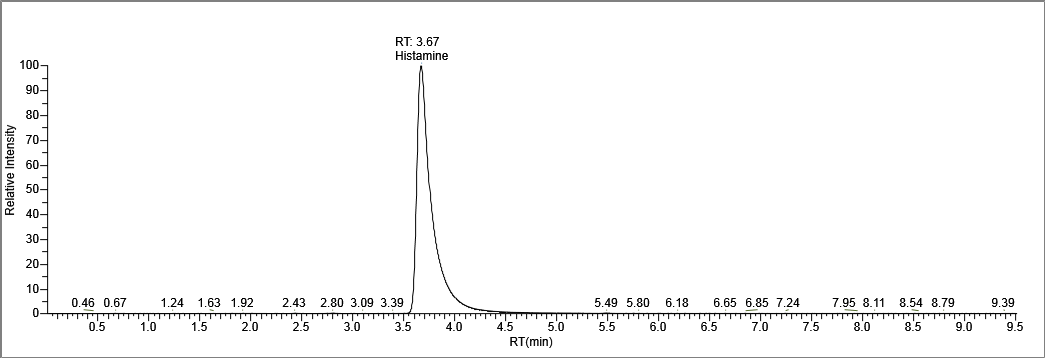


**Mackerel**


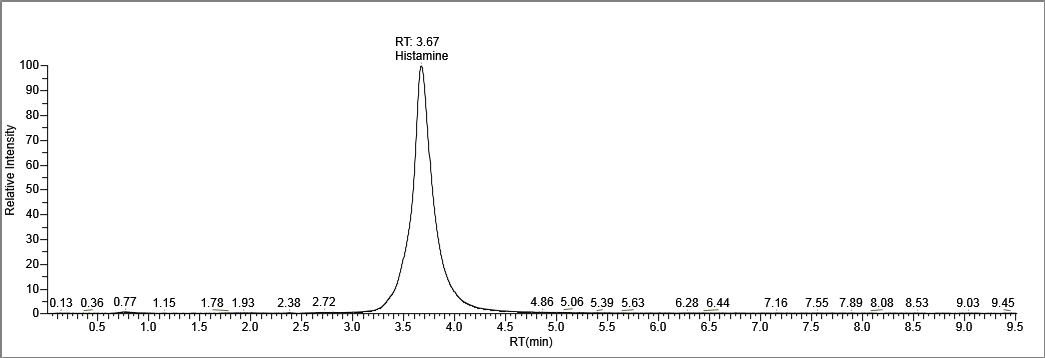


**S3. Data for determination of r% and repeatability ( fish sauce)**

| **Lab Name:** | SAIGONSTC |  |  |  | HISTAMINE |  |  |  |  |  |  |  |
| --- | --- | --- | --- | --- | --- | --- | --- | --- | --- | --- | --- | --- |
| **Instrument:** | Thermo Scientific Instrument |  |  | **Cali File:** | 15.calx |  |  |  |  |  |  |  |
| **User:** | TSQ_Endura |  |  |  |  |  |  |  |  |  |  |  |
| **Batch:** | 15 |  |  |  |  |  |  |  |  |  |  |  |
|  |  |  |  |  |  |  |  |  |  |  |  |  |
|  |  |  |  |  |  |  |  |  |  |  |  |  |
| **Compound Name** | **File Name** | **Sample spike,ppm** | **Calculated  Amt.** | **Sample  wt.** | **Sample  Vol.** | **Dilution Factor** | **Sample  Amt.** | **Units** | **%Rec.** | **Final  report** | SD | %RSD |
| Histamine | R -Fish sauce-1 |  | 347.641 | 1.000 | 50.0 | 20 | 348 | ppm |  | 366 | 24 | 6.6 |
| Histamine | R -Fish sauce 2 |  | 364.905 | 1.000 | 50.0 | 20 | 365 | ppm |  |  |  |  |
| Histamine | R -Fish sauce 3 |  | 387.905 | 1.000 | 50.0 | 20 | 388 | ppm |  |  |  |  |
| Histamine | R -Fish sauce 4 |  | 342.057 | 1.000 | 50.0 | 20 | 342 | ppm |  |  |  |  |
| Histamine | R -Fish sauce 3 |  | 347.544 | 1.000 | 50.0 | 20 | 348 | ppm |  |  |  |  |
| Histamine | R -Fish sauce 6 |  | 408.075 | 1.000 | 50.0 | 20 | 408 | ppm |  |  |  |  |
| Histamine | R -Fish sauce 7 |  | 364.521 | 1.000 | 50.0 | 20 | 365 | ppm |  |  |  |  |
| Histamine | SPIKE-1-IFS18-1 | 214 | 599.255 | 1.000 | 50.0 | 20 | 599 | ppm | 109.0 | 597.6 | 11 | 1.8 |
| Histamine | SPIKE-1-IFS18-2 | 214 | 581.881 | 1.000 | 50.0 | 20 | 582 | ppm | 100.8 |  |  |  |
| Histamine | SPIKE-1--IFS18-3 | 214 | 615.174 | 1.000 | 50.0 | 20 | 615 | ppm | 116.4 |  |  |  |
| Histamine | SPIKE-1--IFS18-4 | 214 | 591.951 | 1.000 | 50.0 | 20 | 592 | ppm | 105.5 |  |  |  |
| Histamine | SPIKE-1--IFS18-5 | 214 | 605.722 | 1.000 | 50.0 | 20 | 606 | ppm | 112.0 |  |  |  |
| Histamine | SPIKE-1--IFS18-6 | 214 | 594.883 | 1.000 | 50.0 | 20 | 595 | ppm | 106.9 |  |  |  |
| Histamine | SPIKE-1-N-IFS18-7 | 214 | 594.344 | 1.000 | 50.0 | 20 | 594 | ppm | 106.7 |  |  |  |
| Histamine | SPIKE-2--IFS18-1 | 428 | 801.343 | 1.000 | 50.0 | 20 | 801 | ppm | 101.7 |  | 20 | 2.55 |
| Histamine | SPIKE-2--IFS18-2 | 428 | 793.317 | 1.000 | 50.0 | 20 | 793 | ppm | 99.8 |  |  |  |
| Histamine | SPIKE-2--IFS18-3 | 428 | 813.272 | 1.000 | 50.0 | 20 | 813 | ppm | 104.5 |  |  |  |
| Histamine | SPIKE-2--IFS18-4 | 428 | 785.83 | 1.000 | 50.0 | 20 | 786 | ppm | 98.1 |  |  |  |
| Histamine | SPIKE-2--IFS18-5 | 428 | 752.123 | 1.000 | 50.0 | 20 | 752 | ppm | 90.2 |  |  |  |
| Histamine | SPIKE-2--IFS18-6 | 428 | 779.227 | 1.000 | 50.0 | 20 | 779 | ppm | 96.5 |  |  |  |
| Histamine | SPIKE-2--IFS18-7 | 428 | 772.326 | 1.000 | 50.0 | 20 | 772 | ppm | 94.9 |  |  |  |
| Histamine | SPIKE-3--IFS18-1 | 856 | 1158.693 | 1.000 | 50.0 | 20 | 1159 | ppm | 92.6 |  | 21 | 1.81 |
| Histamine | SPIKE-3--IFS18-2 | 856 | 1141.615 | 1.000 | 50.0 | 20 | 1142 | ppm | 90.6 |  |  |  |
| Histamine | SPIKE-3--IFS18-3 | 856 | 1134.371 | 1.000 | 50.0 | 20 | 1134 | ppm | 89.8 |  |  |  |
| Histamine | SPIKE-3--IFS18-4 | 856 | 1163.312 | 1.000 | 50.0 | 20 | 1163 | ppm | 93.1 |  |  |  |
| Histamine | SPIKE-3--IFS18-5 | 856 | 1115.814 | 1.000 | 50.0 | 20 | 1116 | ppm | 87.6 |  |  |  |
| Histamine | SPIKE-3--IFS18-6 | 856 | 1137.262 | 1.000 | 50.0 | 20 | 1137 | ppm | 90.1 |  |  |  |
| Histamine | SPIKE-3--IFS18-7 | 856 | 1107.215 | 1.000 | 50.0 | 20 | 1107 | ppm | 86.6 |  |  |  |

| **S4. Compound Calibration Report ( Fist time)** | | | | | | | | | | |
| --- | --- | --- | --- | --- | --- | --- | --- | --- | --- | --- |
|  |  |  |  |  |  |  |  |  |  |  |
| **Lab Name:** | SAIGONSTC | |  | **Method:** | 13-1_HISTAMINE | |  |  |  |  |
| **Instrument:** | Thermo Scientific Instrument | |  |  | HISTAMINE |  |  |  |  |  |
| **User:** | TSQ_Endura | |  | **Cali File:** | 13-1.calx |  |  |  |  |  |
| **Batch:** | 13-1 | |  |  |  |  |  |  |  |  |
|  |  |  |  |  |  |  |  |  |  |  |
|  |  |  | **Compound Name:** | | **Histamine** |  |  |  |  |  |
|  |  |  | 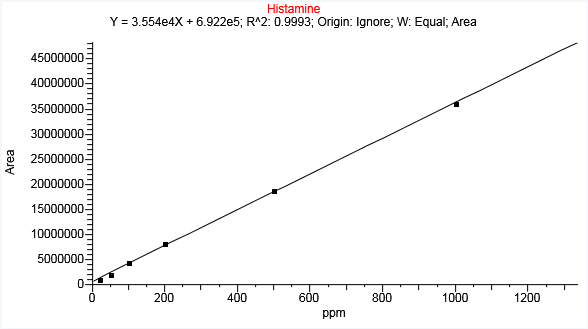   \|  \| \| --- \| |  |  |  |  |  |  |  |
|  |  |  |  |  |  |  |  |  |  |  |
|  |  |  |  |  |  |  |  |  |  |  |
|  |  |  |  |  |  |  |  |  |  |  |
|  |  |  |  |  |  |  |  |  |  |  |
|  |  |  |  |  |  |  |  |  |  |  |
|  |  |  |  |  |  |  |  |  |  |  |
|  |  |  |  |  |  |  |  |  |  |  |
|  |  |  |  |  |  |  |  |  |  |  |
|  |  |  |  |  |  |  |  |  |  |  |
|  |  |  |  |  |  |  |  |  |  |  |
|  |  |  |  |  |  |  |  |  |  |  |
|  |  |  |  |  |  |  |  |  |  |  |
|  |  |  |  |  |  |  |  |  |  |  |
|  |  |  |  |  |  |  |  |  |  |  |
|  |  |  |  |  |  |  |  |  |  |  |
|  |  |  |  |  |  |  |  |  |  |  |
|  |  |  |  |  |  |  |  |  |  |  |
|  |  |  |  |  |  |  |  |  |  |  |
|  |  |  |  |  |  |  |  |  |  |  |
|  |  |  |  |  |  |  |  |  |  |  |
|  |  |  |  |  |  |  |  |  |  |  |
|  |  |  |  |  |  |  |  |  |  |  |
| Linear |  |  |  |  |  |  |  |  |  |  |
| Fail A |  |  |  |  |  |  |  |  |  |  |
| **Level** | **Std Amount** | **Std Area** | **IS Amount** | **IS Area** | **Resp factor/  ratio** | **Calc Amount** | **Units** | **% CV** | **% RSD** |  |
| 1000 | 1000 | 36017140 |  |  | 36017.14 | 994.072 | ppb | N/A | N/A |  |
| 500 | 500 | 18704311 |  |  | 37408.622 | 506.875 | ppb | N/A | N/A |  |
| 200 | 200 | 8279050 |  |  | 41395.25 | 213.499 | ppb | N/A | N/A |  |
| 100 | 100 | 4422298 |  |  | 44222.983 | 104.967 | ppb | N/A | N/A |  |
| 50 | 50 | 2092822 |  |  | 41856.447 | 39.414 | ppb | N/A | N/A | A |
| 20 | 20 | 1089305 |  |  | 54465.239 | 11.174 | ppb | N/A | N/A | A |
